# Biofilm formation onto starch fibres by *Bacillus subtilis* governs its successful adaptation to chickpea milk

**DOI:** 10.1111/1751-7915.13665

**Published:** 2020-10-20

**Authors:** Satish Kumar Rajasekharan, Tali Paz‐Aviram, Shmuel Galili, Zipi Berkovich, Ram Reifen, Moshe Shemesh

**Affiliations:** ^1^ Departmet of Food Science Institute of Postharvvest Technology and Food Sciences Agricultural Research Organization (ARO) The Volcani Center Rishon LeZion 7528809 Israel; ^2^ Department of Vegetable and Field Crops Institute of Plant Sciences Agricultural Research Organization (ARO) The Volcani Center Rishon LeZion 7528809 Israel; ^3^ Institute of Biochemistry, Food Science and Nutrition The Robert H. Smith Faculty of Agriculture, Food and Environment The Hebrew University of Jerusalem Rehovot Israel

## Abstract

Beneficial biofilms may confer effective adaptation to food matrices that assist bacteria in enduring hostile environmental conditions. The matrices, for instance, dietary fibres of various food products, might serve as a natural scaffold for bacterial cells to adhere and grow as biofilms. Here, we report on a unique interaction of *Bacillus subtilis* cells with the resistant starch fibresof chickpea milk (CPM), herein CPM fibres, along with the production of a reddish‐pink pigment. Genetic analysis identified the pigment as pulcherrimin, and also revealed the involvement of Spo0A/SinI pathway in modulating the observed phenotypes. Besides, through successful colonization of the CPM fibres, the wild‐type cells of *B. subtilis* displayed enhanced survivability and resilience to environmental stress, such as heat and *in vitro* gastrointestinal treatments. In total, we infer that the biofilm formation on CPM fibres is an adaptation response of *B. subtilis* for strategic survival.

## Graphical abstract

Cells of probiotic bacterium *Bacillus* *subtilis* interact with the resistant starch fibers of chickpea through preferentially inducing biofilm formation pathway. The successfully colonized starch fibers enable enhanced survivability of the cells during different environmental stresses.

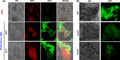

## Introduction

Many bacterial species often exist in most natural settings as matrix‐enclosed sessile cells called biofilm (Vlamakis *et al*., 2013). *Bacillus subtilis* is a beneficial Gram‐positive, spore‐forming bacterium ubiquitously found in soil, gastrointestinal tract (GIT) of ruminants or humans, and food processing plants (Yahav *et al*., 2018). *B. subtilis* inclines to form diverse biofilm phenotypes including pellicle or biofilm bundles in liquid media, and colony‐type biofilm in solid media (Pasvolsky *et al*., 2014; Van Gestel *et al*., 2015). Environmental and physiological conditions often dictate the *B. subtilis* cells for instigating the biofilm mode of growth. The onset of biofilm growth in *B. subtilis* ensues when a specific subset of founder cells triggers the transcription of matrix operons (*epsA‐O,* and *tapA‐sipW‐tasA* operon) for extracellular matrix synthesis via the master regulon, Spo0A (Beauregard *et al*., 2013; Cairns *et al*., 2014). Succinctly, an external signal(s) activates a set of self‐regulating histidine kinases (KinA–KinE), which phosphorylate Spo0A. Phosphorylated Spo0A (Spo0A∼P) governs the transcription of the SinI, a SinR (repressor of matrix genes) antagonist. When Spo0A∼P levels hit the threshold, SinI is actively transcribed that blocks SinR, eventually causing the derepression of matrix genes.

*Bacillus* species belong to versatile microbes that may provide numerous health benefits for the host organism. Some of those species are known to antagonize bacterial pathogens, while others protect or/and promote the growth of probiotic bacteria (Piewngam *et al*., 2018). Also, *Bacillus‐*based probiotics have shown to improve the digestive health by strengthening the intestinal barrier function and by attenuation of the inflammatory response in humans (Rhayat *et al*., 2019). That said, the probiotic candidature of *Bacillus* species is escalating.

Probiotic *Bacillus* species also have a propensity to colonize the human gut transiently (Yahav *et al*., 2018). Furthermore, probiotics can interact with the food matrices, for instance, the non‐digestible fibre portion of the food, which virtually passes intact through the GIT and serve as a scaffold for certain gut microbiota to colonize (Markowiak and Śliżewska, 2017). Thus, probiotics and prebiotics are known to improve human health. A quintessential alternative approach to accentuate health benefits is the coalesced use of probiotics and prebiotics, also known as the synbiotics (Markowiak and Śliżewska, 2017). However, the prospect of probiotics survival in the acidic environment of human GIT is either infrequent or virtually nil. Several studies have been conducted previously to enhance survival of probiotic species in GIT using food matrices (Ranadheera *et al*., 2012; Terpou *et al*., 2017; Terpou *et al*., 2019) through investigating the underlying mechanism(s).

A variety of plant‐based milk is gradually gaining attention due to its copious nutritional values (Sethi *et al*., 2016). It is usually prepared by crushing legumes or nuts with 6–8 volumes of water. Lately, there is substantial interest in developing different kinds of chickpea‐ (*Cicer arietinum*) based milk. Chickpea seeds are rich in healthy nutrients, minerals, proteins, carbohydrates and dietary fibres that are known to favour healthy gut microbiota (Tosh *et al*., 2013; Brüssow, 2020). It also has traditional standards and is a common cuisine in India, middle‐east and the Mediterranean (Gupta *et al*., 2017). The present study was primarily conducted to monitor the biofilm formation by *B. subtilis* in chickpea milk (CPM) and to understand the survival tactics espoused by the bacteria. Through investigating the mechanism of interaction of *B. subtilis* cells with the CPM starch fibres, we discovered intriguing morphological changes enabling the extraordinary adaptability of the cells to growth within CPM.

## Results and discussion

This investigation was initiated for assessing the growth dynamics of *B. subtilis* cells in CPM medium. Initial microscopic analysis (Appendix S1) of the CPM medium revealed the presence of insoluble components that was categorized as non‐fluorescent (Fig. S1A) or autofluorescent fibres (Fig. S1B). Interestingly, the non‐fluorescent fibres of CPM resembled the starch granules (Fig. S1A) in consistent with previous reports (Di Paola *et al*., 2003; Wang *et al*., 2018). Likewise, the presence of autofluorescent starch in dry‐sprayed chickpea seeds was microscopically demonstrated by (Tosh *et al*., 2013). Lugol’s and propidium iodide (PI) staining confirmed that the fibres were indeed starches (Figs S1C and S2). Former stained all of the starch particles in CPM, while the latter selectively stained the autofluorescent starch particles and not the granules, as PI does not penetrate the membranes of the starch granules (Fig. S1C). In addition, we observed an enhanced fibre solubility and loss of autofluorescence at higher pH following the potassium hydroxide (KOH) addition (Fig. S3), thus confirming the autofluorescent fraction as the resistant starch particles.

**Fig. 1:**
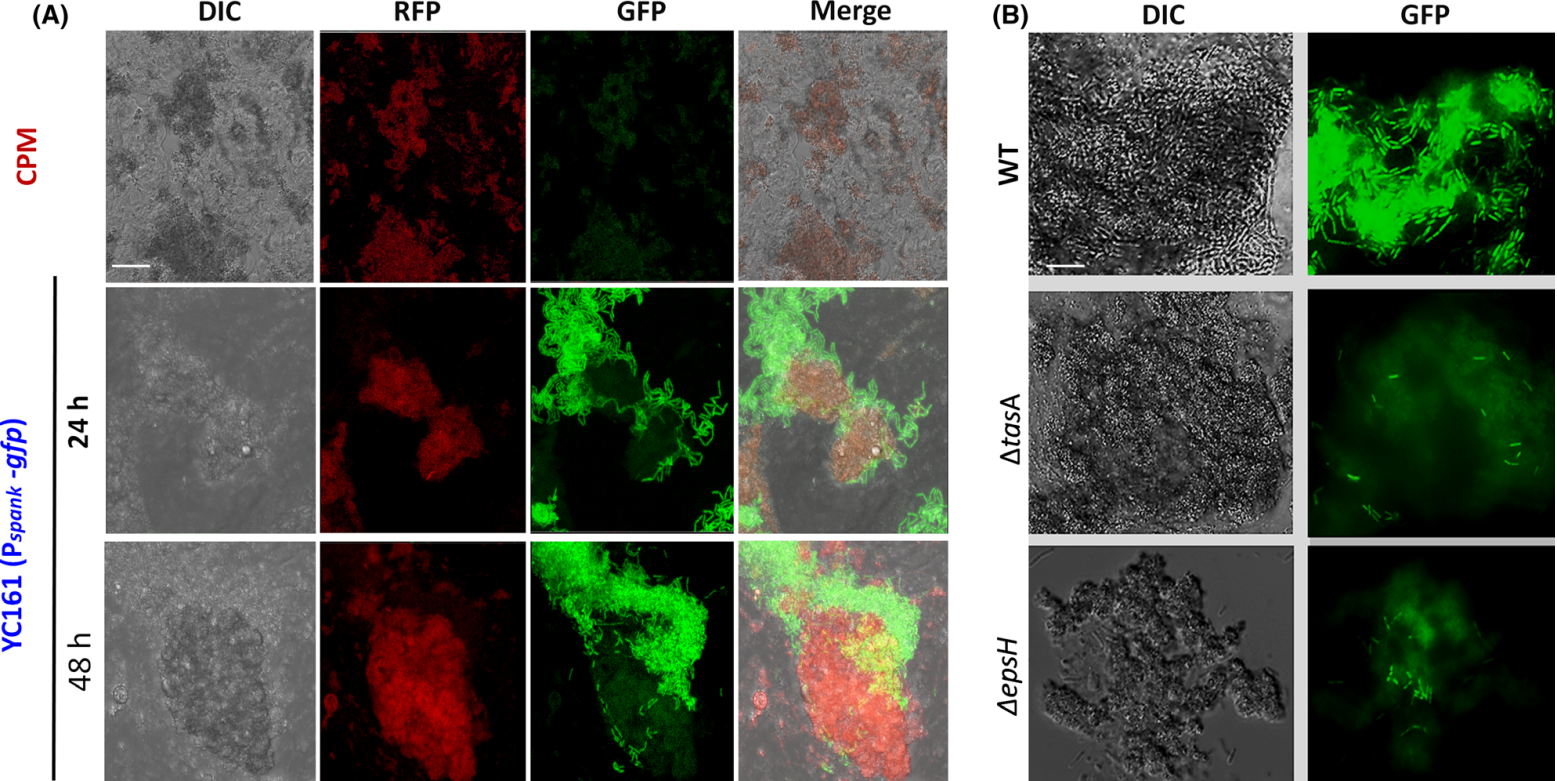
Interaction of *B. subtilis* cells with autofluorescent starch fibres of chickpea milk. A. Z‐sectioning of fluorescently tagged *B. subtilis* cells (YC161) interacting with autofluorescent starch fibres of chickpea milk (CPM). RFP panels show the red autofluorescence of CPM fibres, and GFP panel shows the tagged cells fluorescence, against the faint green autofluorescence of CPM fibres. Scale bar: 20 µm. B. Microscopic imaging of *B. subtilis* WT and matrix mutants (*tasA* and *epsH*) interacting with the CPM fibres, intense green fluorescence is exhibited by the bacteria that was stained with SYTO^TM^ 9 dye and the faint green fluorescence represent the autofluorescence of CPM fibres. Scale bar: 20 µm.

Next, it was investigated whether these starch fibres might serve as a natural scaffold for bacteria to grow as biofilms. To test this, we initially grew fluorescently tagged *B. subtilis* (YC161) in CPM and assessed its ability to interact with these starch fibres (Appendix S1). Interestingly, we found that *B. subtilis* YC161 selectively colonize the autoflouresent starch fibres apparently through tight interactions (Fig. 1A). These intriguing interactions indicate that *Bacillus* cells could preferentially colonize the starch fibres. However, it was noticed that the bacterial cells formed also suspended bundles that did not attach to these fibres and demonstrated extended chaining in CPM (Fig. S4). Z‐stack analysis by confocal laser scanning microscope (CLSM) confirmed that this intriguing bacterial‐fibre interaction was rigid and dynamic (data not shown). We assume that the observed bacterial interactions with colloidal components of CPM might have immense biological and technological importance for the food industry.

Since *B. subtilis* biofilm relies on extracellular polymeric substance matrix, we assessed the growth of strains (*ΔtasA* and *ΔepsH*) that harboured mutants in matrix operons. None of them adhered to the autoflouresent fibres nor formed biofilm bundles in CPM (Fig. 1B), thus affirming the involvement of matrix production in this bacteria‐fibre interaction.

**Fig. 2:**
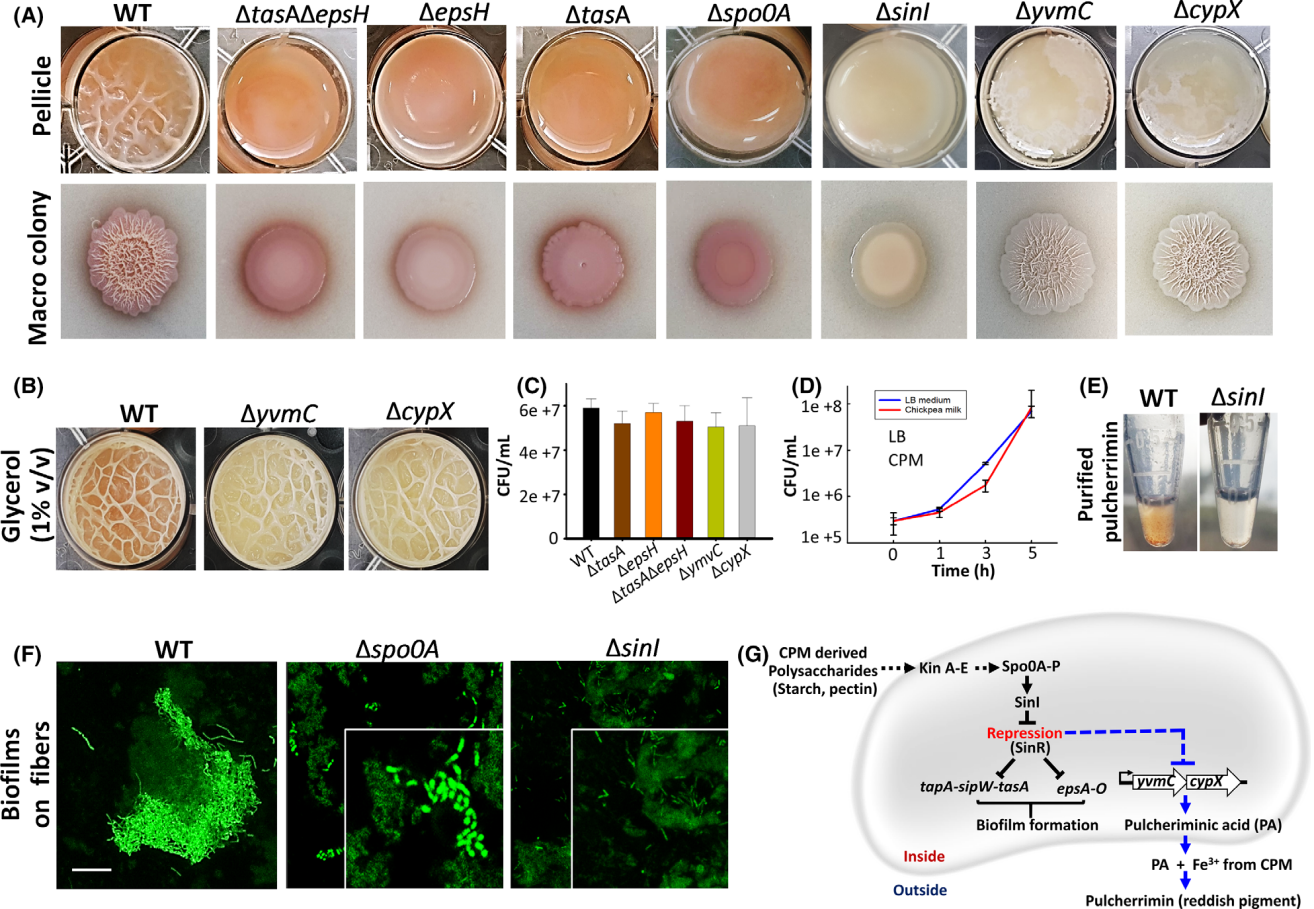
Biofilm formation and pulcherrimin production by *B. subtilis* (WT and mutants cells) grown in chickpea milk. A. Pellicle, pigment and colony‐type biofilm formations by the WT and mutants cells in chickpea milk (CPM) or CPM agar after 72 h of incubation at 30°C. B. Pellicle formations by pulcherrimin‐deficient mutants in CPM in the presence or absence of glycerol (1% v/v) after 72 h of incubation at 30°C. C. Colony forming units (CFUs) of the WT andmutant cells in CPM after 24 h of incubation at 37°C. The graph shows the means ± SEMs of three measurements. *P < 0.05 vs. the non‐treated controls. D. Growth curve of *B. subtilis* WT cells in LB and CPM grown at 37°C with 150 rpm shaking. The graph shows the means ± SEMs of three measurements. E. Purified pulcherrimin (in methanol) from WT, and *sinI* mutants. Detailed processing method is depicted in Fig. S5. F. Interactions of *B. subtilis* WT, *spo0A* and *sinI* mutants (displaying intense green fluorescence) with CPM fibres (faint green autofluorescence). G. A schematic representation depicting the regulation of biofilm formation and pulcherrimin production pathways in *B. subtilis* grown in CPM.

Biofilm forming ability of *B. subtilis* cells was further characterized phenotypically in standing cultures; obviously, the wild‐type (WT) cells formed robust pellicle and colony‐type biofilm in CPM (Appendix S1). As expected, matrix mutants (*ΔtasA, ΔepsH* and the double mutant) could not form either type of biofilm in CPM (Fig. 2A). Since matrix mutants failed to form a biofilm, we assumed that the observed phenomenon could be related to the Spo0A/SinI regulatory pathway. We show that the strains harbouring deletion mutations in either *spo0A* or *sinI* did not form pellicle or colony biofilm, nor attachment to the starch fibres (Fig. 2A and F). The best possible explanation of the observed results is that in the absence of SinI, SinR represses the biofilm phenotypes in CPM. However, in an unprecedented occurrence, *Δspo0A* mutants formed eccentric cell clusters or bunches in CPM, while not adhering to the starch fibres(Fig. 2F). We postulate that in the absence of Spo0A, cells might activate a different yet unknown pathway that triggers undefined cell clustering. Involvement of the possible alternative pathway(s) other than the Spo0A/SinI need to be substantiated in future studies.

We further observed that the WT cells of *B. subtilis* produced an inquisitive reddish‐pink pigmentation in CPM (Fig. 2A). The pigmentation was also observed in *epsH*, *tasA* and *spo0A* mutants (Fig. 2A). Intriguingly, the *sinI* mutant cells refrained from producing the pigment. *B. subtilis* provides an assorted spectrum of pigments that may or may not be correlated with biofilm formation (Arnaouteli *et al*., 2019). Pulcherrimin is one such pigment that *B. subtilis* produces during biofilm mode of growth (Arnaouteli *et al*., 2019). Pigment production by *Bacilli* is also recognized as a survival tactics exhibited by the vegetative cells against external stressors. Since the biochemical traits of pulcherrimin were close to the observed pigment (Fig. S5), and that the pulcherrimin biosynthesis pathway in *B. subtilis* was well established (Arnaouteli *et al*., 2019), we first checked this pathway using pulcherrimin‐deficient mutants. The mutants lacked the enzymes, YvmC (Cyclo(l‐leucyl‐l‐leucyl) synthase) and CypX (Pulcherriminic acid synthase), that convert two tRNA‐ molecules of leucine to pulcherriminic acid (PA), which in turn binds to iron (Fe^3+^) to form pulcherrimin. The mutants failed to produce the pigment in solid and liquid CPM (Fig. 2A), confirming our perception. It was also reported that pulcherrimin could be induced by the addition of glycerol or starch (Li *et al*., 2017). When tested in CPM, glycerol apparently induced robustness in the pellicles, although it did not enhance the qualitative pigmentation levels in WT strain (Fig. 2B). However, the glycerol failed to induce the pigmentation in pulcherrimin‐deficient strains (Fig. 2B). Consequently, we think that the pigment is indeed pulcherrimin, but it remains unclear the necessity of its production by *B. subtilis* cells in CPM. One possible explanation would be that the pulcherrimin production in CPM might be attributed to bacterial‐starch interactions, suggesting starch might be a carbon source for triggering this phenotype. That said, pulcherrimin is also shown to possess powerful antimicrobial effectiveness (Türkel and Ener, 2009; Kántor *et al*., 2019). Several studies suggested improving the yield of this pigment in strains that indigenously produce pulcherrimin, or identifying the media that enhances it. The CPM and other chickpea‐based food products could be an ideal influential medium for producing high yields of pulcherrimin. Also, since certain *B. subtilis* strains demonstrate antagonistic activity against food‐borne pathogens (Moore *et al*., 2013; Sipiczki, 2020), the production of pulcherrimin in CPM by those strains would further potentiate their probiotic traits.

Next, pulcherrimin was further purified as previously described (Kluyver *et al*., 1953) and compared between WT and *sinI* mutant. The results showed no pigment production in the *ΔsinI* mutant (Fig. 2E). Although precarious, we presume pigmentation has firm reciprocity with the biofilm phenotypes potentially related to SinI regulatory protein (Fig. 2G). The growth of all the tested strains had no significant change (Fig. 2C), and the growth curve of WT in CPM was analogous to its growth in Lysogeny broth (LB) in shaking cultures (Fig. 2D). The results suggest that all the tested mutants displayed defects in matrix synthesis without hampering the growth in CPM; whereas the *ΔsinI* mutant showed a further deficiency in the pulcherrimin synthesis. An additional challenge was identifying the signalling molecule (presumably carbon source) that might be responsible for the biofilm or pigment formation. Our going on observations indicate that the soluble fibre, pectin and no other tested tested sugars (glucose, fructose, sucrose and raffinose) are involved in biofilm and pigment formation (Fig. S6).

**Fig. 3:**
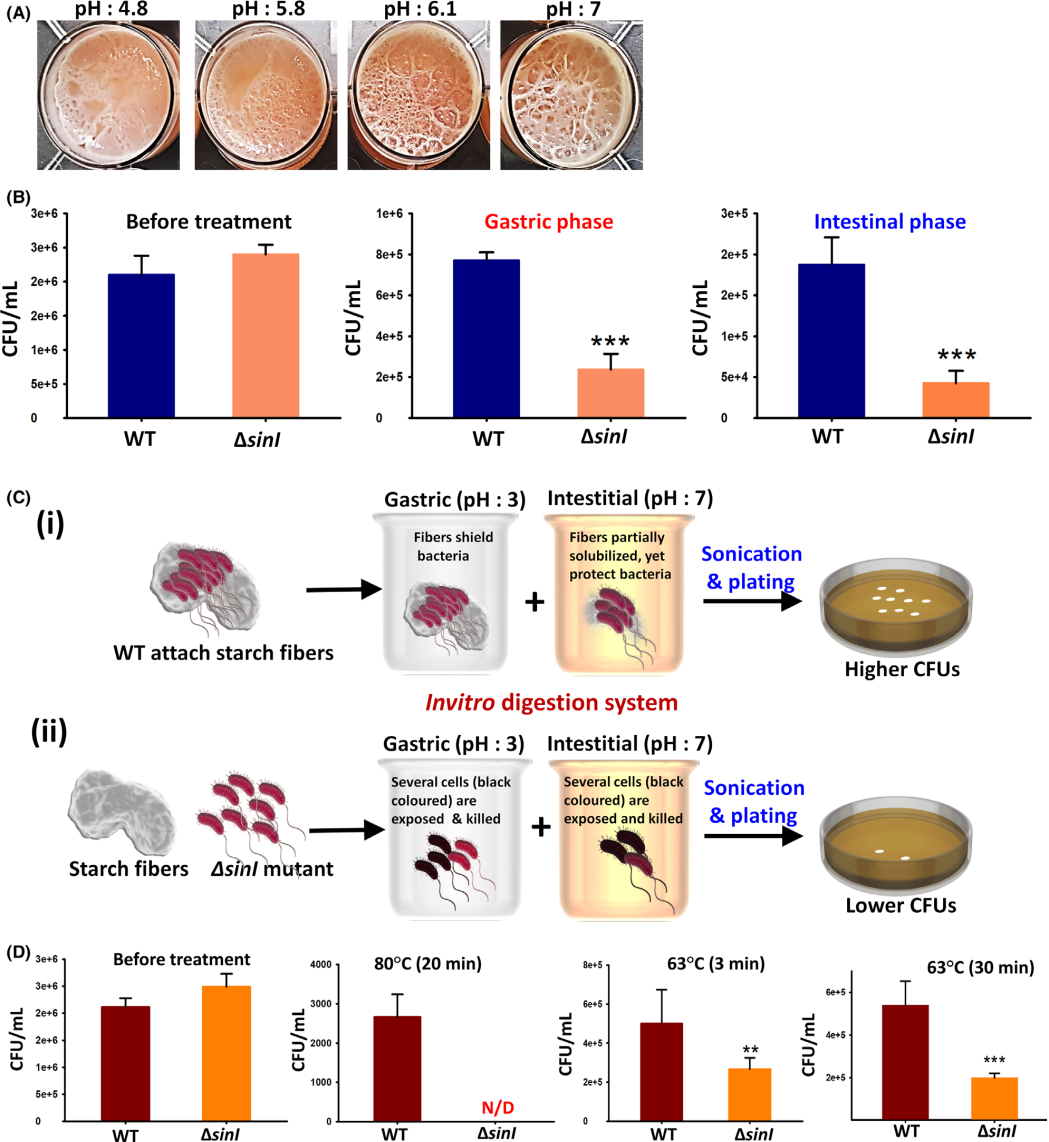
Survivability of *B. subtilis* following pasteurization and *in vitro* gastrointestinal digestion. A. Pellicle formation of *B. subtilis* WT cells in chickpea milk (CPM) adjusted to different pH and incubated at 30°C for 72 h. B. Colony forming units (CFUs) of *B. subtilis* WT and *sinI* mutant, after subjecting them to *in vitro* gastrointestinal digestion, and sonication (2 min (10s pulse on/off) at 4°C with 40% amplitude). The graph shows the means ± SEMs of three measurements. ** *P* < 0.01, and *** *P* < 0.001 vs. the non‐treated controls. C. Schematic model describing the bacterial‐fibre interactions and their surviva in gastric and intestinal phases of *in vitro* digestion system. Detailed experimental procedure is depicted in Fig. S8. D. Effect of *B. subtilis* WT and *sinI* mutant to heat pasteurization at various temperatures and time durations. The graph shows the means ± SEMs of three measurements. ** *P* < 0.01, and *** *P* < 0.001 vs. the non‐treated controls, N/D: Not detected.

Our further investigation was aspired to understand the biological relevance of bacterial‐fibre interactions in CPM. The purpose is to inspire the use of CPM as a dietary supplement enriched with probiotics. CPM could serve as a natural source for prebiotics, which are the microbiome‐shaping components that provide the carbon source for the beneficial microbes in the human gut. These fibrespass through the gastrointestinal tract virtually intact and undigested. In the colon, they are utilized by gut microbiota, which digests them to distribute nutrients to the colonic epithelium, thus maintaining a functional and healthier digestive system (Rhayat *et al*., 2019). Enriching CPM with probiotics will generate a blend of the probiotic‐prebiotic complex. The complex might help probiotics during transit through the acidic gastric environment without being killed, considering prebiotics might protect them and allow fast passage through the GIT. We first tested the ability of the WT strain to grow in different pHs. Alkaline pH seemed to enhance the robustness in the pellicle formation, while pigment was better produced at lower pH (Fig. 3A). Conversely, if probiotics are supplemented alone, they might seldom survive the acidic environment of the GIT (Kimelman and Shemesh, 2019). To prove this hypothesis, we first assessed the viability of WT strain grown in LB medium following *in vitro* digestion system as described previously (Yahav *et al*., 2018) (Appendix S1). We noticed a significant loss of viability of cells grown in LB compared with cells grown in CPM (Fig. S7). WT cells grown in CPM were resilient to hostile effects during *in vitro* digestion system. They exhibited higher survival rates than *ΔsinI* mutants in both gastric and intestinal phases (Fig. 3B, and Fig. S7). Higher bacterial survival rates in CPM transcends to its ability to interact with the fibres. We have previously shown that *B. subtilis* forms biofilm bundles, which can be estimated by the CFU method, following proper sonication that liberates the cells from the bundles (Ben‐Ishay *et al*., 2017). Additionally, similar assays were conducted by other groups (Hemmatian and Kim, 2019) to quantify the bacteria adhered to porous, multi‐layered fibres. Similarly, in this study, we used sonication to liberate the bacterial cells that adhered to the chickpea fibres, as shown in the flowchart (Fig. S8). Further, we tested the survival rates of *ΔsinI* mutants that existed as individual cells in CPM. Interestingly, we observed a drastic reduction in the viability of *ΔsinI* mutant confirming the potential role of CPM starch fibres in protecting the bacteria, as depicted in Figure 3B and C.

Milk and dairy‐free milk products like CPM need to undergo thermal processing like pasteurization before packaging. Probiotic cells are sensitive to heat and can hardly endure this procedure (Lahtinen, 2012). Microencapsulation techniques have shown to improve the viability and are often recommended (Elshaghabee *et al*., 2017). Here, we anticipated that the prebiotic fibres in CPM might shield bacteria from physical stresses such as heat treatment (Appendix S1). Consequently, the WT cells showed remarkable survivability, while the amount of the *ΔsinI* mutant cells was significantly reduced following exposure to heat treatment (Fig. 3D). Higher survival rates in the WT strain ascribe to its ability to form biofilms on the starch fibres that, in turn, shield it from heat stress. The *ΔsinI* mutant that existed as free cells in CPM was highly susceptible to heat treatments. Overall, we emphasize that the interaction of *B. subtilis* cells with starch fibres in CPM provides ideal conditions to withstand varied stressors.

The fibre content in chickpea is considerably high, similarly to other legume‐based non‐dairy milk, which includes almond and soybean (Niño‐Medina *et al*., 2019). Fibre‐rich diets are known to reduce the risks associated with diabetes and heart diseases (de Camargo *et al*., 2019). The insoluble fibres in chickpea are known to treat/prevent constipation and supports weight management. It was also shown that dietary fibres are responsible for transporting dietary antioxidants through the GIT (Saura‐Calixto, 2011). Here, we demonstrate yet another essential function of the chickpea fibres that function as a scaffold for biofilm formation, thus providing protective clothing for the probiotic bacteria to persevere the acidic environment in the GIT.

## Conclusions

Various types of plant‐based milk are often favoured over dairy milk as it offers a comprehensive nutritional package. Among them, CPM is gaining immense popularity due to its multifarious health benefits (Li *et al*., 2016). The current study further establishes CPM as an enriched media that potentially favours the growth of the probiotic bacteria, such as *B. subtilis*, which might open new directions for studying other bacterial or yeast probiotics. Another important finding of autofluorescent starch fibre in CPM imparts dynamic scaffolding properties for the probiotic bacteria to attach and flourish. Besides, the biofilm formation phenomenon of *B. subtilis* in CPM is still open for future research, though we partially uncover the involvement of Spo0A/SinI signal pathway and show that the SinI antirepressor is vital for biofilm and pulcherrimin formations in CPM. Lastly, we demonstrate the resilience of *B. subtilis* to hostile conditions appears to be credited to the tight molecular interactions with the starch fibres. In total, these findings emphasize the adaptative responses acquired by bacteria for their strategic survival.

## Conflict of interest

The authors have no conflict of interest to declare.

## Supporting information


**Table S1.** *B. subtilis* strains used in this study.
**Fig. S1.** Microscopic visualization and staining of the chickpea starch fibers. Non‐fluorescent starch granules (A), auto‐fluorescent starch fibers that are ruptured due to heat during autoclaving (B), scale bar: 20 µm, and comparative analysis of natural autofluorescence and propidium iodide staining (C). Propidium iodide stains the starch fibers that are ruptured, while not the intact starch granules as the dye is membrane impermeable. For analysis, unvarying minimal auto‐exposure (20 ms) was used which sinks out the natural autoflouresence of CPM, scale bar: 100 µm.
**Fig. S2.** Lugol's stain (potassium iodide and iodine) confirms that most of the CPM is starch. KI stained CPM (A), and microscopic visualization shows insoluble fibers as blue color (indication of starch, as KI is orange colored and turns dark blue if it binds to starch) under light microscope (B). Yellow arrow denotes the auto‐fluorescent starch fibers while the black arrow and all that shows faint blue is the non‐fluorescent starch granules.
**Fig. S3.** Alkaline pH (by addition of KOH to CPM) solubilizes the starch fibers as well as quenches the autofluorescence, thus confirming them as the resistant starch fibers.
**Fig. S4.** Extensive chaining in CPM by WT strains. GFP expression was monitored in case of the fluorescently tagged *B. subtilis* (YC161), while the untagged NCIB3610 strain was stained with SYTO^TM^ 9 dye.
**Fig. S5.** Sequential biochemical procedure for extraction of pulcherrimin from CPM.
**Fig. S6.** CPM polysaccharides act as an environmental signal for biofilm formation. Most of the carbon sources tested formed fragile pellicle in LB supplemented with 0.1 mM manganese (A), while pectin (the only soluble dietary fiber in chickpea) formed induced biofilm (as pellicle as well as colony type biofilm) at 0.1% and 0.5% concentration (B and C).
**Fig. S7.** Survivability of *B. subtilis* following *in vitro* gastro‐intestinal digestion. Quantitation of *B. subtilis* survival was done based on colony forming units (CFU) of WT cells grown in either LB or CPM at 30 °C (with shaking at 25 rpm for 24 h), following to the either gastric or intestinal phases of the *in vitro* gastro‐intestinal digestion.
**Fig. S8.** Diagrammatic representation illustrating the detailed experimental procedures for analysis of the CPM fiber‐attached bacteria following pasteurization and *in vitro*‐digestion system.
**Appendix S1.** Experimental procedure
