# Supplementary material for: Biofilm formation onto starch fibres by Bacillus subtilis governs its successful adaptation to chickpea milk
Source: Microb Biotechnol. 2020 Oct 20;14(4):1839–46. doi: 10.1111/1751-7915.13665 (PMC8313274; doi:10.1111/1751-7915.13665)
Supplement: Supplementary file 1 — Table S1. B. subtilis strains used in this study. Fig. S1. Microscopic visualization and staining of the chickpea starch fibers. Non‐fluorescent starch granules (A), auto‐fluorescent starch fibers that are ruptured due to heat during autoclaving (B), scale bar: 20 µm, and comparative analysis of natural autofluorescence and propidium iodide staining (C). Propidium iodide stains the starch fibers that are ruptured, while not the intact starch granules as the dye is membrane impermeable. For analysis, unvarying minimal auto‐exposure (20 ms) was used which sinks out the natural autoflouresence of CPM, scale bar: 100 µm. Fig. S2. Lugol's stain (potassium iodide and iodine) confirms that most of the CPM is starch. KI stained CPM (A), and microscopic visualization shows insoluble fibers as blue color (indication of starch, as KI is orange colored and turns dark blue if it binds to starch) under light microscope (B). Yellow arrow denotes the auto‐fluorescent starch fibers while the black arrow and all that shows faint blue is the non‐fluorescent starch granules. Fig. S3. Alkaline pH (by addition of KOH to CPM) solubilizes the starch fibers as well as quenches the autofluorescence, thus confirming them as the resistant starch fibers. Fig. S4. Extensive chaining in CPM by WT strains. GFP expression was monitored in case of the fluorescently tagged B. subtilis (YC161), while the untagged NCIB3610 strain was stained with SYTOTM 9 dye. Fig. S5. Sequential biochemical procedure for extraction of pulcherrimin from CPM. Fig. S6. CPM polysaccharides act as an environmental signal for biofilm formation. Most of the carbon sources tested formed fragile pellicle in LB supplemented with 0.1 mM manganese (A), while pectin (the only soluble dietary fiber in chickpea) formed induced biofilm (as pellicle as well as colony type biofilm) at 0.1% and 0.5% concentration (B and C). Fig. S7. Survivability of B. subtilis following in vitro gastro‐intestinal digestion. Quantitation of B. subtilis [file MBT2-14-1839-s001.doc]

**Supporting information**

**Biofilm formation onto starch fibers by *Bacillus subtilis* governs its successful adaptation to chickpea milk**

Satish Kumar Rajasekharan^1^, Tali Paz^1^, Shmuel Galili^2^, Zipi Berkovich^3^, Ram Reifen^3^, and Moshe Shemesh^1^*

^1^Department of Food Science, Institute of Postharvest Technology and Food Sciences, Agricultural Research Organization (ARO), The Volcani Center, Rishon LeZion 7528809, Israel.

^2^ Department of Vegetable and Field Crops, Institute of Plant Sciences, Agricultural Research Organization (ARO), The Volcani Center, Rishon LeZion 7528809, Israel.

^3^Institute of Biochemistry, Food Science and Nutrition, The Robert H. Smith Faculty of Agriculture, Food and Environment, The Hebrew University of Jerusalem, Rehovot, Israel

***Corresponding author:** moshesh@agri.gov.il

**Table S1. *B. subtilis* strains used in this study.**

| **Strain** | **Genotype** | | **Reference** |
| --- | --- | --- | --- |
| *B. subtilis* NCIB3610 | Wild-type strain | (Branda, et al., 2001) | |
| *B. subtilis* YC161 | P*_spank-_gfp* in 3610, CM^R^ | (Chai, et al., 2011) | |
| *B. subtilis* DI103 | *ΔepsHΔtasA* in 3610 | Shemesh M lab | |
| *B. subtilis* RL3852 | *ΔepsH* in 3610 | (Branda, et al., 2006) | |
| *B. subtilis* SB505 | *ΔtasA* in 3610 | (Kearns, et al., 2005) | |
| *B. subtilis* CY211 | *ΔsinI* in 3610 | (Pasvolsky, et al., 2014) | |
| *B. subtilis* RL4620 | *Δspo0A* in 3610 | (Shemesh and Chai, 2013) | |
| *B. subtilis* NRS5532 | *ΔcypX* in 3610 | (Arnaouteli, et al., 2019) | |
| *B. subtilis* NRS5533 | *ΔyvmC* in 3610 | (Arnaouteli, et al., 2019) | |
|  |  |  | |
|  |  |  | |
|  |  |  | |
|  |  |  | |
|  |  |  | |
|  |  |  | |
|  |  |  | |
|  |  |  | |
|  |  |  | |
|  |  |  | |


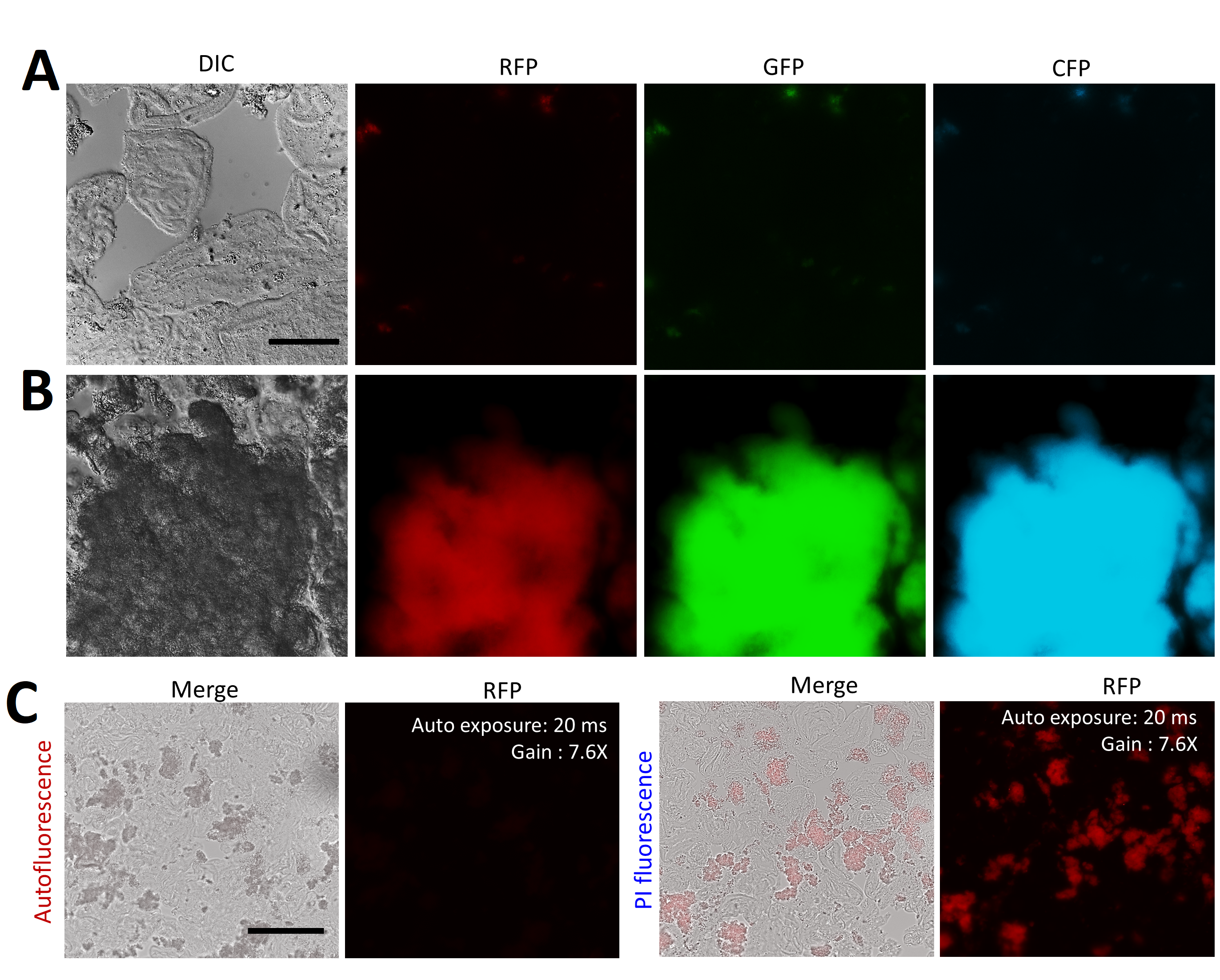


**Fig. S1. Microscopic visualization and staining of the chickpea starch fibers.** Non-fluorescent starch granules (A), auto-fluorescent starch fibers that are ruptured due to heat during autoclaving (B), scale bar: 20 µm, and comparative analysis of natural autofluorescence and propidium iodide staining (C). Propidium iodide stains the starch fibers that are ruptured, while not the intact starch granules as the dye is membrane impermeable. For analysis, unvarying minimal auto-exposure (20 ms) was used which sinks out the natural autoflouresence of CPM, scale bar: 100 µm.
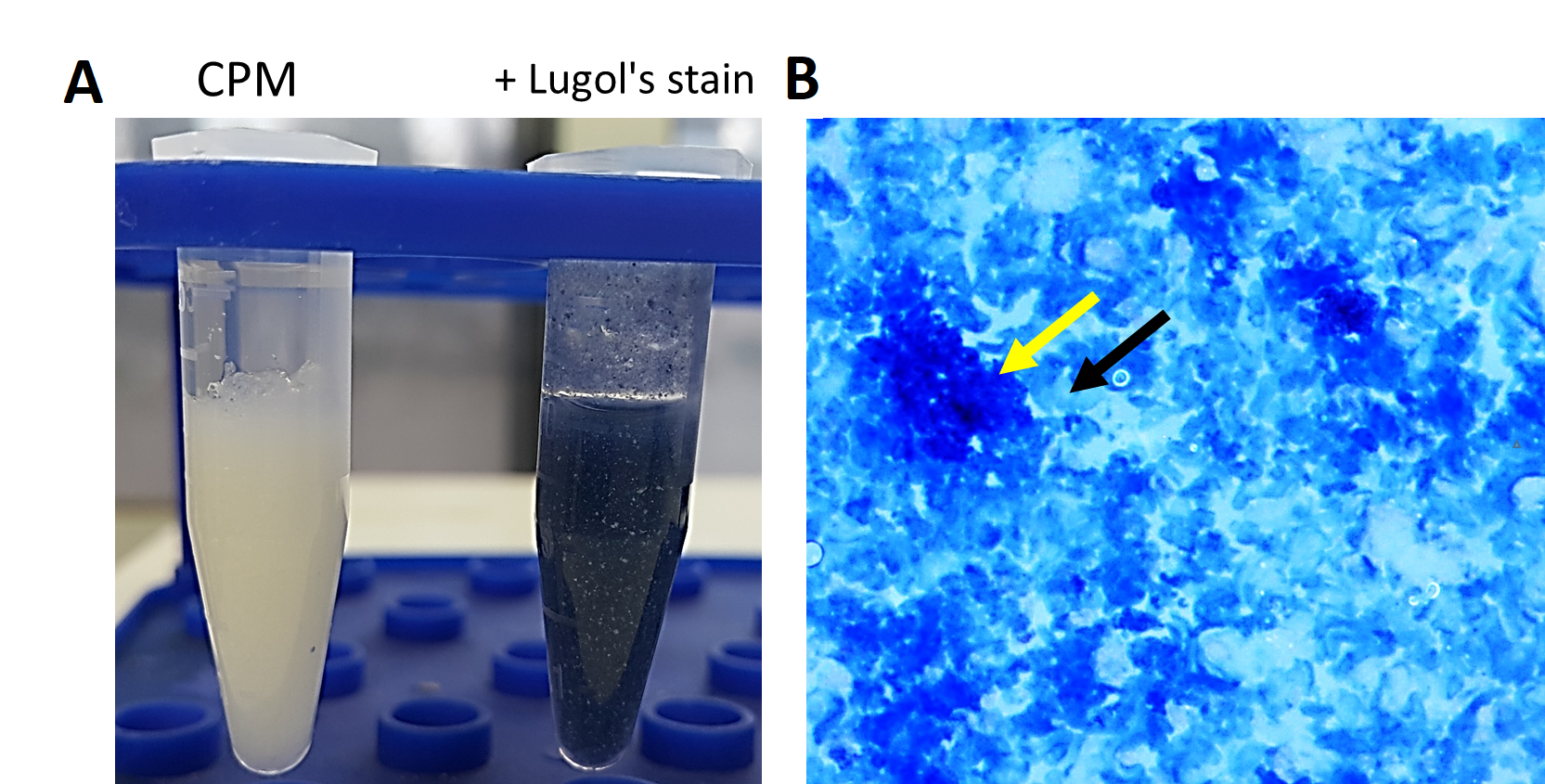


**Fig. S2. Lugol's stain (potassium iodide and iodine) confirms that most of the CPM is starch.** KI stained CPM (A), and microscopic visualization shows insoluble fibers as blue color (indication of starch, as KI is orange colored and turns dark blue if it binds to starch) under light microscope (B). Yellow arrow denotes the auto-fluorescent starch fibers while the black arrow and all that shows faint blue is the non-fluorescent starch granules.

**
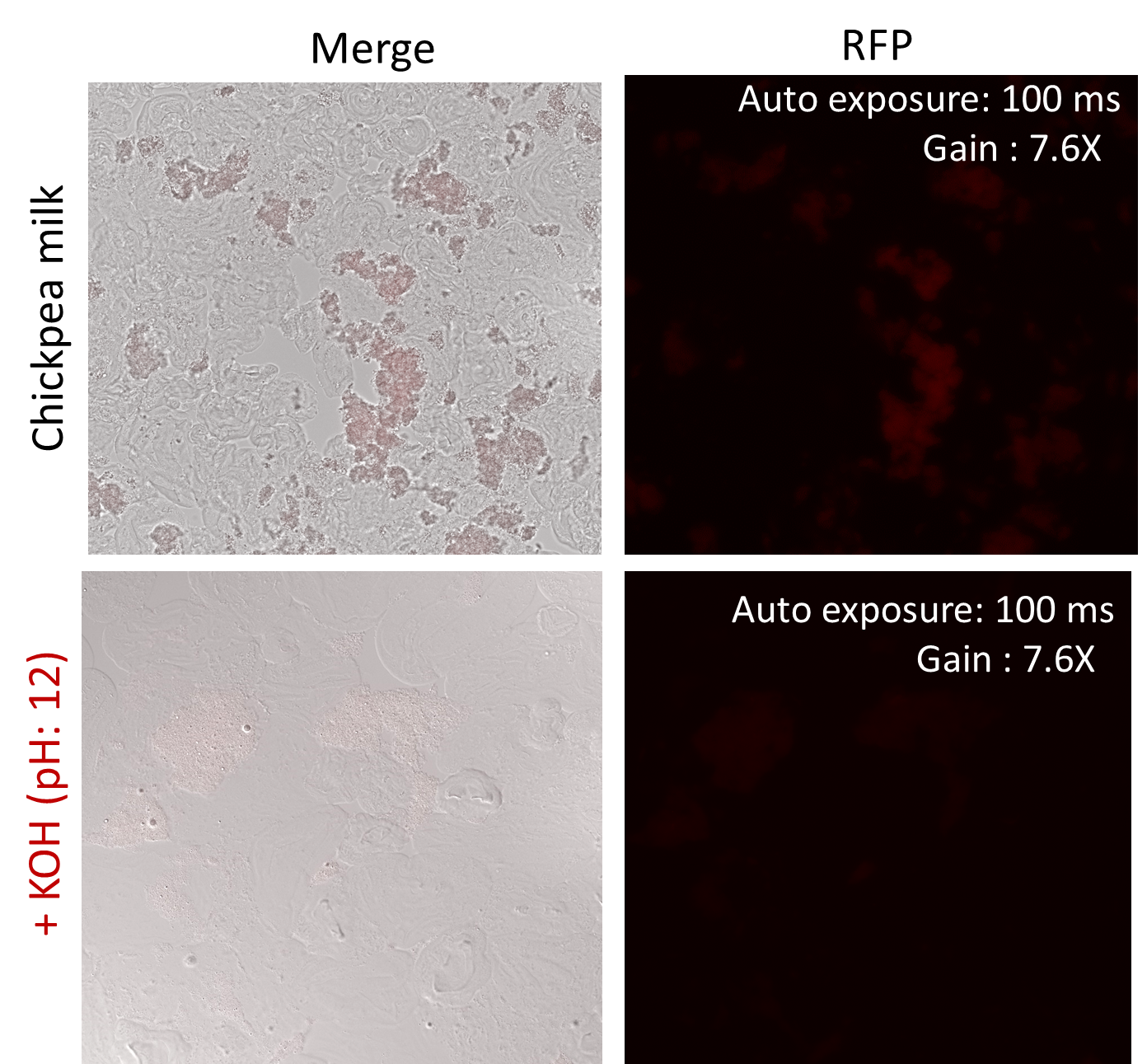
**

**Fig. S3. Alkaline pH (by addition of KOH to CPM) solubilizes the starch fibers as well as quenches the autofluorescence, thus confirming them as the resistant starch fibers.**


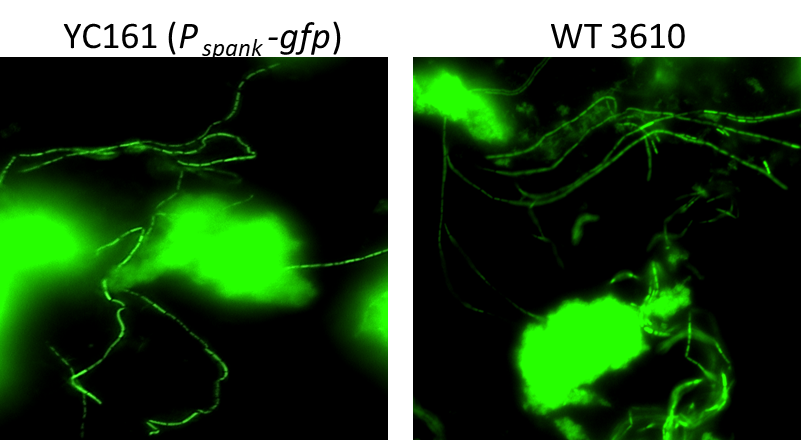


**Fig. S4. Extensive chaining in CPM by WT strains.** GFP expression was monitored in case of the fluorescently tagged *B. subtilis* (YC161), while the untagged NCIB3610 strain was stained with SYTO^TM^ 9 dye.

**
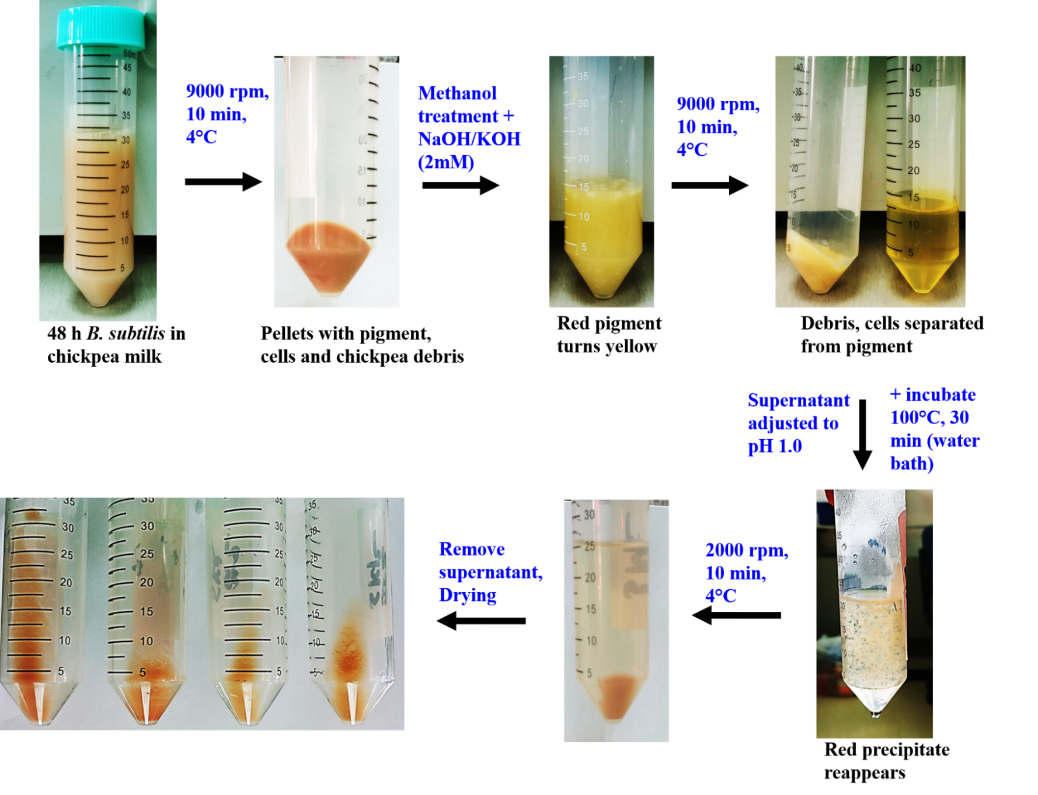
**

**Fig. S5. Sequential biochemical procedure for extraction of pulcherrimin from CPM.**

**
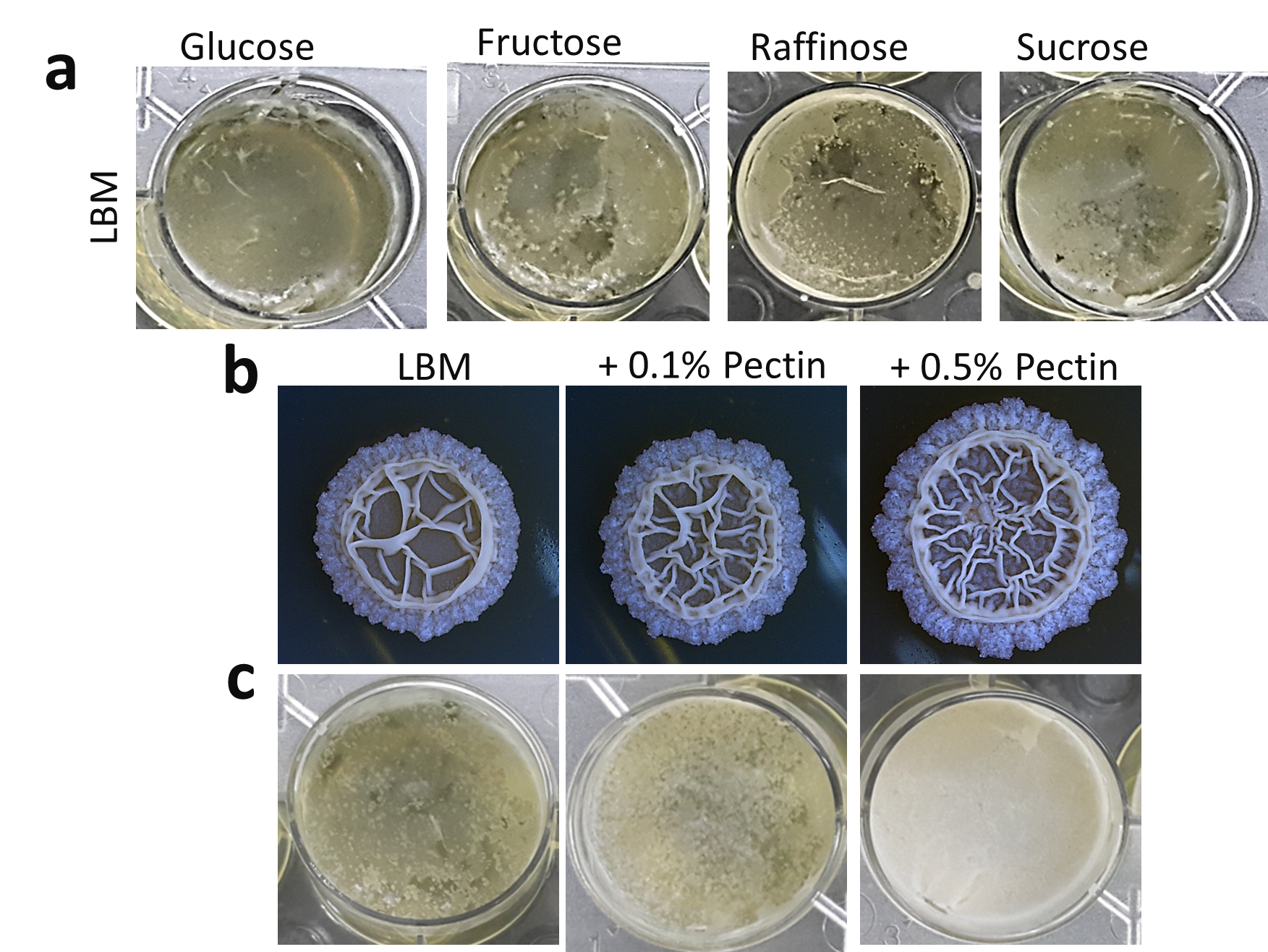
 Fig. S6. CPM** **polysaccharides act as an environmental signal for biofilm formation.** Most of the carbon sources tested formed fragile pellicle in LB supplemented with 0.1 mM manganese (A), while pectin (the only soluble dietary fiber in chickpea) formed induced biofilm (as pellicle as well as colony type biofilm) at 0.1% and 0.5% concentration (B and C).


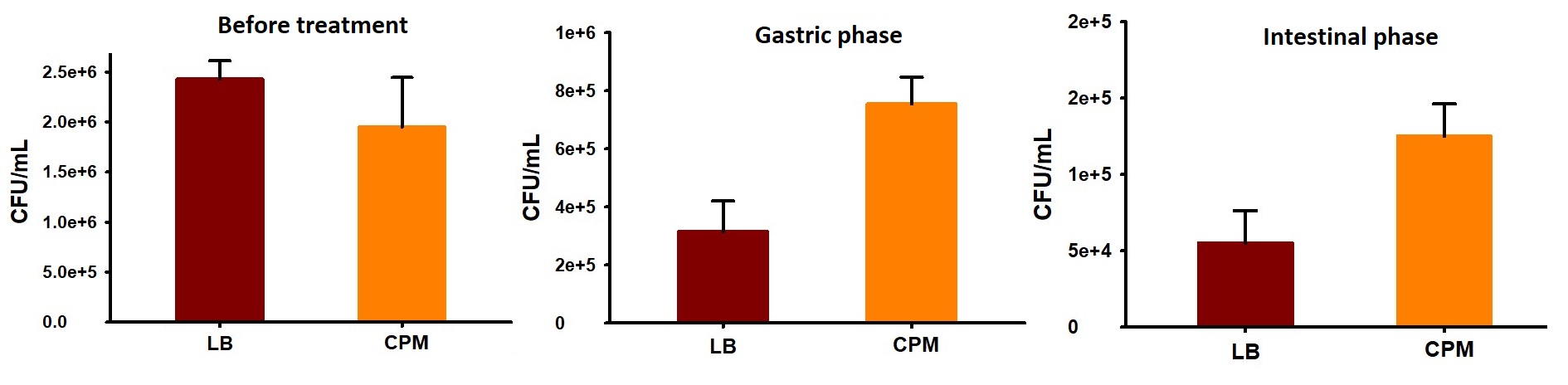


**Fig. S7. Survivability of *B. subtilis* following *in vitro* gastro-intestinal digestion.** Quantitation of *B. subtilis* survival was done based on colony forming units **(**CFU) of WT cells grown in either LB or CPM at 30 °C (with shaking at 25 rpm for 24 h), following to the either gastric or intestinal phases of the *in vitro* gastro-intestinal digestion.


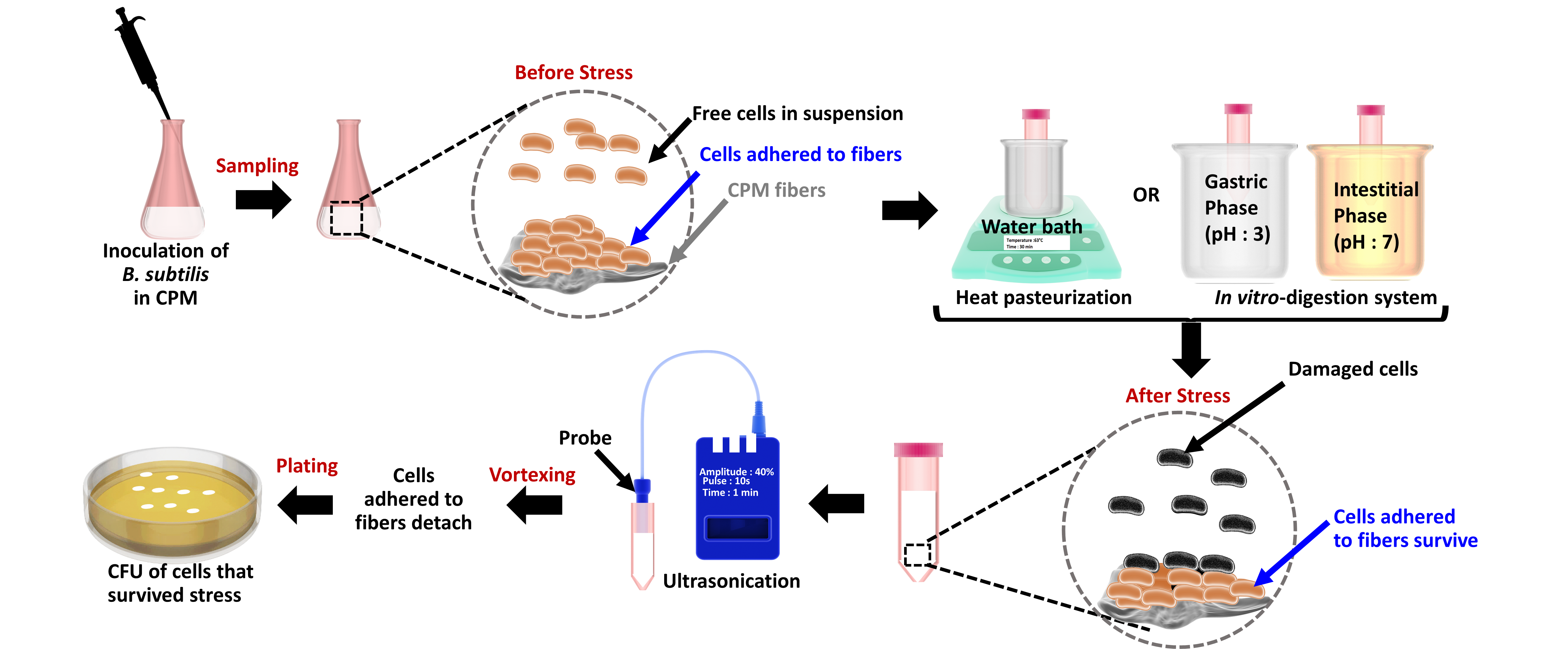
**Fig. S8. Diagrammatic representation illustrating the detailed experimental procedures for analysis of the CPM fiber-attached bacteria following pasteurization and *in vitro*-digestion system.**

**Appendix S1**

**Experimental procedure**

**Bacterial strains and culture conditions**

*B. subtilis* strains used in this study are listed in S1 Table. All strains were cultured in Lysogeny broth (LB) comprising 10 g of tryptone, 5 g of yeast extract, and 5 g of NaCl (per 1 liter of medium), or on solid LB medium supplemented with 1.5% agar. For generating the starter cultures, initially, the strains were streaked on LB agar plates from the glycerol stocks maintained at -80 ⁰C and incubated overnight at 37°C. A colony from the overnight LB agar plate was inoculated in fresh LB and grown at 37°C for 5h (through shaking at 150 rpm). The resultant culture was used as the starter culture for the subsequent experiments.

**Chickpea milk preparation**

Approximately, 187.5 g of Kabuli-type chickpea (*Cicer arietinum*) seed were soaked in 1.2 L of distilled water (DW) and incubated for 12 h at room temperature. Following incubation, fresh DW was replaced and the seeds were crushed using a blender. The liquid chickpea seed suspension, herein chickpea milk (CPM) was boiled for 10 min, filtered using a sieve, following which the pH was adjusted with lemon juice (for pH: 4.8, 5.8 and 6.1) or 1 mM NaOH (for pH: 7). Finally, CPM was autoclaved and stored for further assays.

**Macroscopic assessment of biofilms**

Starter cultures of the required strains were prepared as previously described. For pellicle formation assays, 5 μl of the bacterial suspensions (5 x 10^5^ CFU/mL) were pipetted into 4 mL of CPM in a 12 well polystyrene plates, while for the colony-type biofilm assays, 3 μl of the bacterial suspensions were spotted on a CPM solid medium supplemented with 1.5% agar. All the plates were incubated at 30°C for 72 h and images were captured using either a regular camera or a Zeiss Stemi 2000-C microscope with an axiocam ERc 5s camera (Zeiss, Germany).

**Microscopic analysis**

For visualization of CPM components, propidium iodide (Promega, USA) and Lugol’s stain was used, while for visualization of *B. subtilis* interactions with the CPM fibers, fluorescently tagged B. subtilis YC161 strain, that constitutively expressed the green fluorescent protein (GFP) was used. The samples were proceeded and stained as previously described (Pasvolsky, et al., 2014) and visualized under a confocal laser scanning microscope (CLSM) (Leica, Wetzler, Germany). Strains that did not express GFP were stained with SYTO^TM^ 9 dye from the Filmtracer live/dead biofilm viability kit (Promega, USA) in conformity with the guidelines.

***In vitro* digestion system**

The survivability of *B. subtilis* WT and *ΔsinI* mutants to acidic environment was monitored by *in vitro* digestion system (Yahav, et al., 2018). Briefly, the starters were diluted 1:100 into LB or CPM and incubated at 30 °C with shaking at 25 rpm for 24h, following which the samples were subjected to *in vitro* gastro-intestinal digestion procedures as previously described (Piewngam, et al., 2018). Following *in vitro* digestion, samples were sonicated for 2 min (10s pulse on/off) at 4°C with 40% amplitude, plated on LB agar plates and incubated overnight at 37°C. Following incubation, the surviving cells (that attach the CPM fibers) were enumerated by colony forming units (CFU) method (Hemmatian and Kim, 2019).

**Assessment of bacterial sensitivity to heat treatment**

The sensitivity of the *B. subtilis* WT and *sinI* mutant cells to heat treatment was monitored by pasteurization. Starter cultures of the bacterial strains were prepared as previously described. The cultures were then diluted 1:100 in CPM and grown for 24 h at 30°C with 25 rpm shaking. Following incubation, the samples were then heat treated in three separate batches or tubes (63°C for 3 min, 63°C for 30 min, and 80°C for 20 min) in a water bath. Immediately after pasteurization, the samples were sonicated for 2 min (10s pulse on/off) at 4°C with 40% amplitude with an Ultrasonic processor (Sonics, VCX 130, Newtown, USA), diluted, plated and incubated overnight at 37°C. The number of surviving cells were enumerated by the CFU method.

**Statistical analysis**

All experiments were conducted in triplicate, and results are expressed as means ± standard deviations. Statistical significance was determined by pair-wise testing using the Students’ *t*-test, and was accepted for *p* values of **p* <0.05, ** *p<*0.01, and *** *p<*0.001.

**References**

Arnaouteli, S., Matoz-Fernandez, D., Porter, M., Kalamara, M., Abbott, J., MacPhee, C.E., et al. (2019) Pulcherrimin formation controls growth arrest of the *Bacillus subtilis* biofilm, *PNAS* **116**: 13553-13562.

Branda, S.S., Chu, F., Kearns, D.B., Losick, R., and Kolter, R. (2006) A major protein component of the *Bacillus subtilis* biofilm matrix, *Mol. Microbiol.* **59**: 1229-1238.

Branda, S.S., González-Pastor, J.E., Ben-Yehuda, S., Losick, R., and Kolter, R. (2001) Fruiting body formation by *Bacillus subtilis*, *Proceedings of the National Academy of Sciences* **98**: 11621-11626.

Chai, Y., Norman, T., Kolter, R., and Losick, R. (2011) Evidence that metabolism and chromosome copy number control mutually exclusive cell fates in *Bacillus subtilis*, *The EMBO Journal* **30**: 1402-1413.

Hemmatian, T., and Kim, J. (2019) Quantification methods for textile-adhered bacteria: extraction, colorimetric, and microscopic analysis, *Polymers* **11**: 1666.

Kearns, D.B., Chu, F., Branda, S.S., Kolter, R., and Losick, R. (2005) A master regulator for biofilm formation by *Bacillus subtilis*, *Mol. Microbiol.* **55**: 739-749.

Pasvolsky, R., Zakin, V., Ostrova, I., and Shemesh, M. (2014) Butyric acid released during milk lipolysis triggers biofilm formation of *Bacillus* species, *Int. J. Food Microbiol* **181**: 19-27.

Piewngam, P., Zheng, Y., Nguyen, T.H., Dickey, S.W., Joo, H.-S., Villaruz, A.E., et al. (2018) Pathogen elimination by probiotic *Bacillus* via signalling interference, *Nature* **562**: 532-537.

Shemesh, M., and Chai, Y. (2013) A combination of glycerol and manganese promotes biofilm formation in *Bacillus subtilis* via histidine kinase KinD signaling, *J. Bacteriol.* **195**: 2747-2754.

Yahav, S., Berkovich, Z., Ostrov, I., Reifen, R., and Shemesh, M. (2018) Encapsulation of beneficial probiotic bacteria in extracellular matrix from biofilm-forming *Bacillus subtilis*, *Artif Cells Nanomed Biotechnol* **46**: 974-982.
